# Trypanosoma brucei Homologue of Regulator of Ribosome Synthesis 1 (Rrs1) Has Direct Interactions with Essential Trypanosome-Specific Proteins

**DOI:** 10.1128/mSphere.00453-19

**Published:** 2019-08-07

**Authors:** Daniel Jaremko, Martin Ciganda, Noreen Williams

**Affiliations:** a Department of Microbiology and Immunology, University at Buffalo, Buffalo, New York, USA; Carnegie Mellon University

**Keywords:** 5S rRNA, Rpf2, Rrs1, protein-RNA, protein-protein, ribosome biogenesis

## Abstract

Studies in eukaryotic ribosome biogenesis have largely been performed in yeast, where they have described a highly complex process involving numerous protein and RNA components. Due to the complexity and crucial nature of this process, a number of checkpoints are necessary to ensure that only properly assembled ribosomes are released into the cytoplasm. Assembly of the 5S ribonucleoprotein (RNP) complex is one of these checkpoints for late-stage 60S subunit maturation. Studies in Saccharomyces cerevisiae have identified the 5S rRNA and four proteins, L5, L11, Rpf2, and Rrs1, as comprising the ribosome-associated 5S RNP. Work from our laboratory has shown that in the eukaryotic pathogen Trypanosoma brucei, the 5S RNP includes trypanosome-specific proteins P34/P37, as well as homologues of L5, Rpf2, and 5S rRNA. In this study, we examine a homologue of Rrs1 and identify it as an additional member of the T. brucei 5S RNP. Using RNA interference, we show that TbRrs1 is essential for the survival of T. brucei and has an important role in ribosome subunit formation and, together with TbRpf2, plays a role in 25/28S and 5.8S rRNA processing. We further show that TbRrs1 is a member of the T. brucei 5S RNP through the identification of novel direct interactions with P34/P37 and 5S rRNA as well as with TbL5 and TbRpf2. These unique characteristics of TbRrs1 highlight the importance of studying ribosome biogenesis in the context of diverse organisms and identify interactions that could be targeted for future drug development.

**IMPORTANCE** Trypanosoma brucei is a parasite responsible for human and animal African trypanosomiasis. Current treatments for these diseases have numerous problems, and the development of novel chemotherapeutics can be achieved by identifying targets that are parasite specific and part of essential processes. Ribosome biogenesis is the process of generating translation-competent ribosomes and is critical for survival in all organisms. Work from our laboratory has shown that the formation of the 5S RNP, a crucial checkpoint in ribosome biogenesis, requires trypanosome-specific proteins P34/P37 and homologues of Rpf2 and L5 which possess parasite-specific characteristics. In this study, we characterize TbRrs1, an additional member of the T. brucei 5S RNP, and show that it is essential for parasite survival and has unique interactions with P34/P37 and 5S rRNA. This expands our understanding of the 5S RNP in T. brucei and identifies new targets for future drug development.

## Abstract (precis)

Trypanosoma brucei is a parasite responsible for human and animal African trypanosomiasis. Current treatments for these diseases have numerous problems, and the development of novel chemotherapeutics can be achieved by identifying targets that are parasite specific and part of essential processes. Ribosome biogenesis is the process of generating translation-competent ribosomes and is critical for survival in all organisms. Work from our laboratory has shown that the formation of the 5S RNP, a crucial checkpoint in ribosome biogenesis, requires trypanosome-specific proteins P34/P37 and homologues of Rpf2 and L5 which possess parasite-specific characteristics. In this study, we characterize TbRrs1, an additional member of the T. brucei 5S RNP, and show that it is essential for parasite survival and has unique interactions with P34/P37 and 5S rRNA. This expands our understanding of the 5S RNP in T. brucei and identifies new targets for future drug development.

## INTRODUCTION

Ribosome biogenesis in Saccharomyces cerevisiae involves 4 rRNAs, 90 ribosomal proteins, and more than 200 assembly factors (1). These components are shuttled to nascent ribosomes and incorporated at distinct steps to allow for essential conformational shifts and recruitment of other components. Disruption of this process leads to a loss of translation-competent ribosomes. Checkpoints are necessary to ensure that only properly assembled ribosomes are released into the cytoplasm. Assembly and incorporation of the 5S ribonucleoprotein complex (5S RNP), a protein-RNA neighborhood found in the 60S subunit, is one of these checkpoints (2). Studies of the 5S RNP from S. cerevisiae have shown that the 5S RNP contains 5S rRNA, ribosomal proteins L5 and L11, and assembly factors Rrs1 and Rpf2. Interrupting incorporation of any of these members disrupts assembly of the 5S RNP into the 60S subunit and this branch of ribosome biogenesis (2).

While studies in yeast have provided insight into the complexity of ribosome biogenesis, there is still limited information about the differences in this process in other eukaryotes. Trypanosoma brucei is a eukaryotic parasite and the causative agent of human African trypanosomiasis (HAT) and animal African trypanosomiasis (AAT), which are diseases that pose devastating economic and health burdens for sub-Saharan African countries where they are endemic (3). Current treatments for HAT and AAT suffer from extreme adverse side effects and developing resistance (4–6). One way to identify promising new drug targets is to characterize parasite-specific components that are critical to essential pathways such as ribosome biogenesis.

Studies of ribosome biogenesis in T. brucei, while limited, have highlighted a number of conserved and divergent features. Among these are the presence of the trypanosome-specific proteins P34/P37 as unique members of the 5S RNP. Loss of P34/P37 in T. brucei results in a lethal phenotype, a disruption of 60S subunit maturation (7), and a decrease of 5S rRNA abundance. The role for P34/P37 in the 5S RNP (7) is supported by data showing that these proteins bind 5S rRNA *in vivo* (8) and *in vitro* (9) and that P34 interacts *in vivo* and *in vitro* with the T. brucei homologues of L5 (10) and Rpf2 (11). However, nothing is known about the T. brucei homologue of Rrs1. In S. cerevisiae, Rrs1 is the binding partner of Rpf2, forming a tight heterodimer to complete the biogenesis of ribosomes in *Xenopus* (BRIX) RNA-binding domain in Rpf2 (12–15). S. cerevisiae Rrs1 (ScRrs1) is essential, with a role in 25S rRNA and 60S subunit maturation (16), and is required for the *in vitro* stability of Rpf2. ScRrs1 also directly interacts with the proteins Rpf2 (17), L11 (18), and L5 (2). However, the significance of T. brucei Rrs1 (TbRrs1) in ribosome biogenesis (TriTryp accession number Tb927.6.2050) has not been addressed.

Our laboratory had previously determined that TbRpf2 was a member of the T. brucei 5S RNP with trypanosome-specific characteristics. Since little is known about Rrs1 beyond the yeast model system, we were interested in examining TbRrs1 and how its role in the 5S RNP might differ in the context of the trypanosome-specific components. We first assessed changes in cell survival and morphology upon loss of TbRrs1. We then analyzed protein levels of other members of the 5S RNP to determine whether they were impacted by the loss of TbRrs1. We next assessed the role of TbRrs1 in ribosome biogenesis by analyzing the abundance of ribosomal subunits as well as changes in rRNA processing. Finally, a series of *in vivo* and *in vitro* assays were performed to determine which members of the 5S RNP interact with TbRrs1.These studies expanded our understanding of the 5S RNP beyond the model organism S. cerevisiae and allowed us to identify features of this highly conserved process that differ between diverse eukaryotic organisms, potentially allowing for the future development of trypanocidal drugs.

## RESULTS

### TbRrs1 is essential for T. brucei growth and survival.

**FIG 1:**
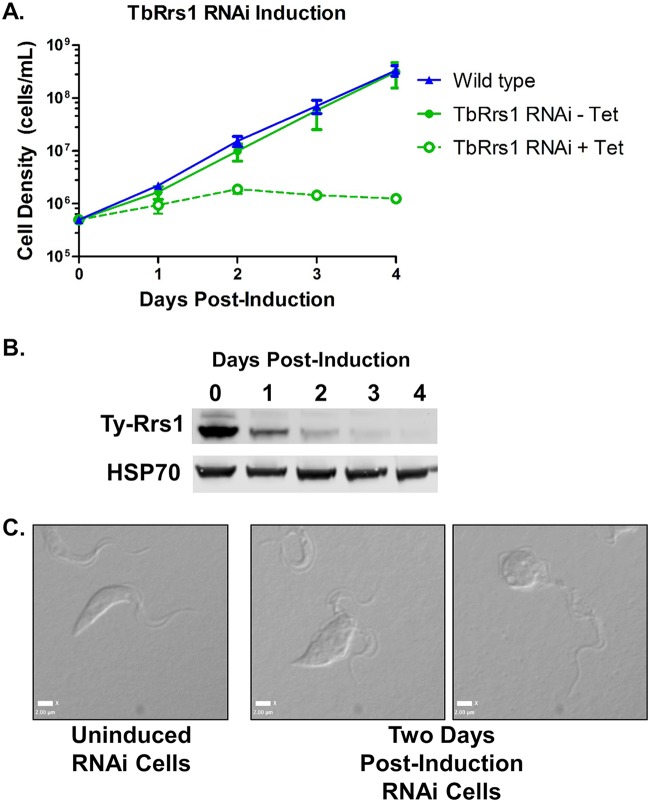
TbRrs1 is an essential protein for T. brucei survival. (A) Growth curves comparing wild-type cells (blue line) versus uninduced and induced TbRrs1 RNAi cells (green lines). (B) Western blot analysis was performed on cell extracts collected at days 0 to 4 with or without induction with HSP70 as a loading control. (C) DIC microscopy images of cells taken with or without 2 days of RNA interference. Analyses were performed on three biological replicates, and representative images are shown.

We developed cell lines containing a 10×-Ty tag incorporated into one copy of the TbRrs1 gene in both a wild-type and an RNA interference (RNAi) background for inducible knockdown of TbRrs1. While the uninduced RNAi line grew comparably to wild-type cells, induction of RNAi and resulting loss of TbRrs1 significantly impaired both growth and survival (Fig. 1A). Knockdown resulted in a decrease in TbRrs1 to 0.16 (standard deviation [SD], 0.09) (relative to uninduced cells) after day 1 of induction and a decrease to 0.02 (SD, 0.02) by day 3 (Fig. 1B). After 2 days, the cells became enlarged and multiflagellated or condensed to small round shapes (Fig. 1C). Together, these results show that TbRrs1 is essential in T. brucei.

### Loss of TbRrs1 results in a decreased abundance of TbRpf2.

**FIG 2:**
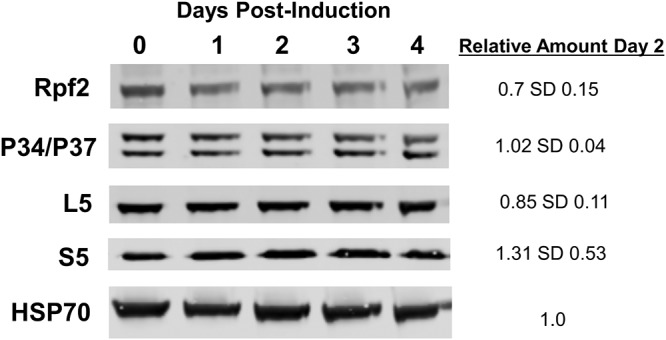
Loss of TbRrs1 results in a slightly decreased abundance of TbRpf2. Western blot analysis was performed using cell extracts collected at days 0 to 4 with or without RNAi induction. Antibodies used to probe for each specific protein are indicated beside each blot, with HSP70 used as a loading control. Analyses were performed on three biological replicates, average values with standard deviations were calculated, and representative blots are shown.

We next examined the impact that knockdown had on the other protein members of the 5S RNP at 2 days postinduction. Loss of TbRrs1 did not significantly impact the levels of TbL5 (0.85 [SD, 0.11], relative to uninduced cells) or P34/P37 (1.02 [SD, 0.04]). However, we did see a mild decrease in levels of TbRpf2 to 0.70 (SD, 0.15) at 2 days postinduction (Fig. 2). Furthermore, although the results were variable, there was an increase in S5 protein levels (1.31 [SD, 0.53]), perhaps suggesting an impact on 40S subunit proteins (Fig. 2). Taken together, this shows that while TbRrs1 is essential for survival, the loss of TbRrs1 impacts levels of its binding partner TbRpf2 but not the other 5S RNP proteins.

### Depletion of TbRrs1 disrupts 60S subunit biogenesis and decreases active translation.

**FIG 3:**
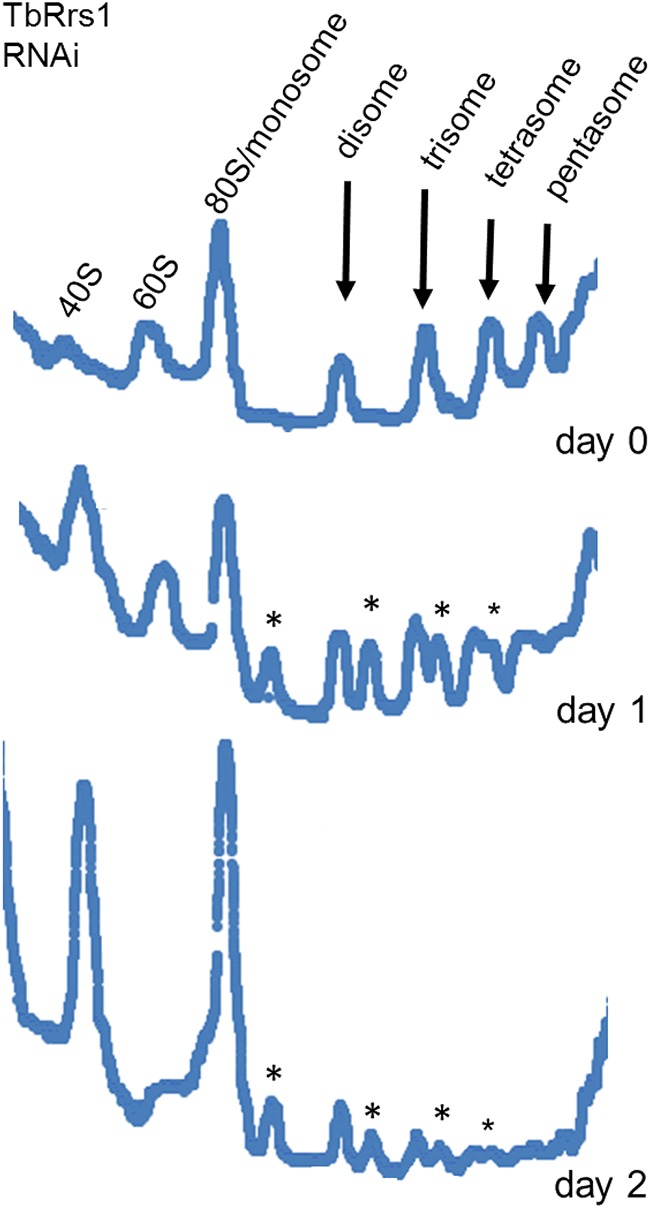
Depletion of TbRrs1 disrupts 60S subunit biogenesis and decreases active translation. Cells were collected at days 0 to 2 after induction of RNA interference knockdown of TbRrs1, and polysome profile analyses were performed on extracts from these cells. Arrows indicate polysome peaks, and asterisks indicate halfmer peaks. Analyses were performed on three biological replicates, and representative tracings are shown.

We investigated the effect that loss of TbRrs1 has on levels of ribosomal subunits, monosomes (80S particle), and polysomes. We found a progressive increase in the 40S/60S ratio, suggesting a deficiency in the assembly of mature 60S particles, or decreased stability of assembled 60S particles. In addition, polysomal peaks decreased as induction of the RNAi progressed (Fig. 3, arrows). Most notably, a decrease in levels of TbRrs1 leads to the appearance of peaks of intermediate density between particles containing *n* ribosomes and particles containing (*n* + 1) ribosomes. These peaks of intermediate density are instances of defects in subunit joining or 60S subunit biogenesis, such that the 60S subunit is unable to bind to the 40S subunit/mRNA complex. These “halfmers,” which have been described and characterized elsewhere (19), can be detected on sedimentation on a sucrose gradient (Fig. 3, asterisks).

### 25/28S and 5.8S rRNA processing are disrupted by loss of TbRrs1 or TbRpf2.

**FIG 4:**
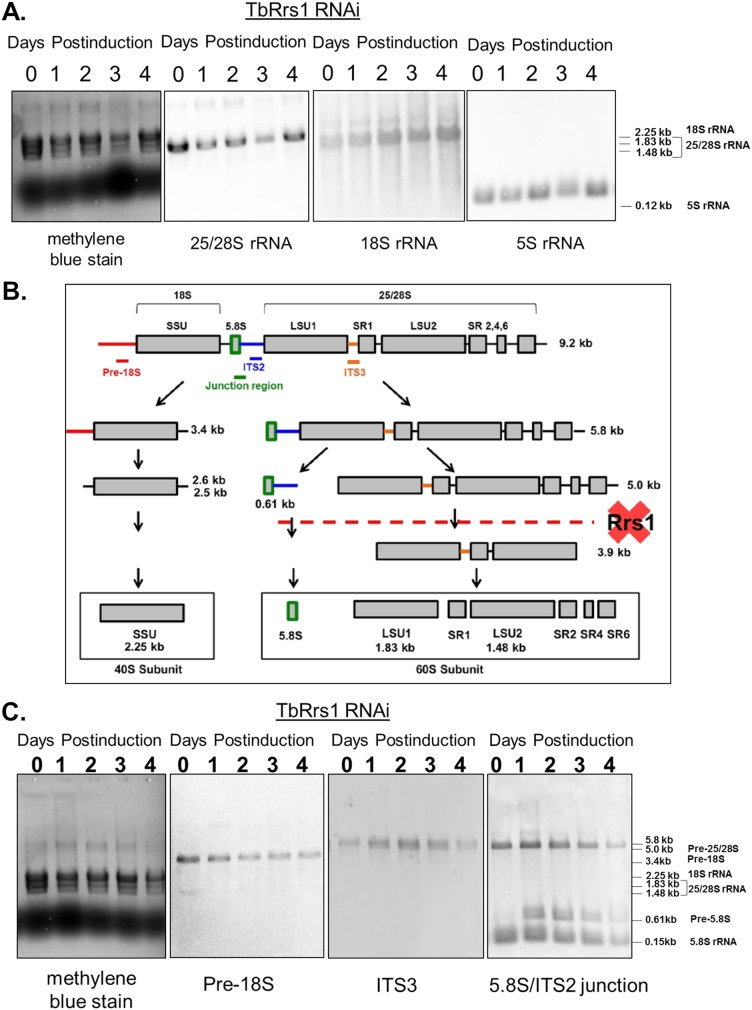
25/28S and 5.8S rRNA processing are disrupted by loss of TbRrs1. Total RNA was extracted from cells at days 0 to 4 of RNA interference induction as indicated. RNA was stained with methylene blue and then probed for various mature rRNAs (A) or rRNA processing intermediates (C) as labeled. Panel B is a map of rRNA processing altered to show the proposed site of processing interruption (38). Analyses were performed on three biological replicates, and representative blots are shown.

**FIG 5:**
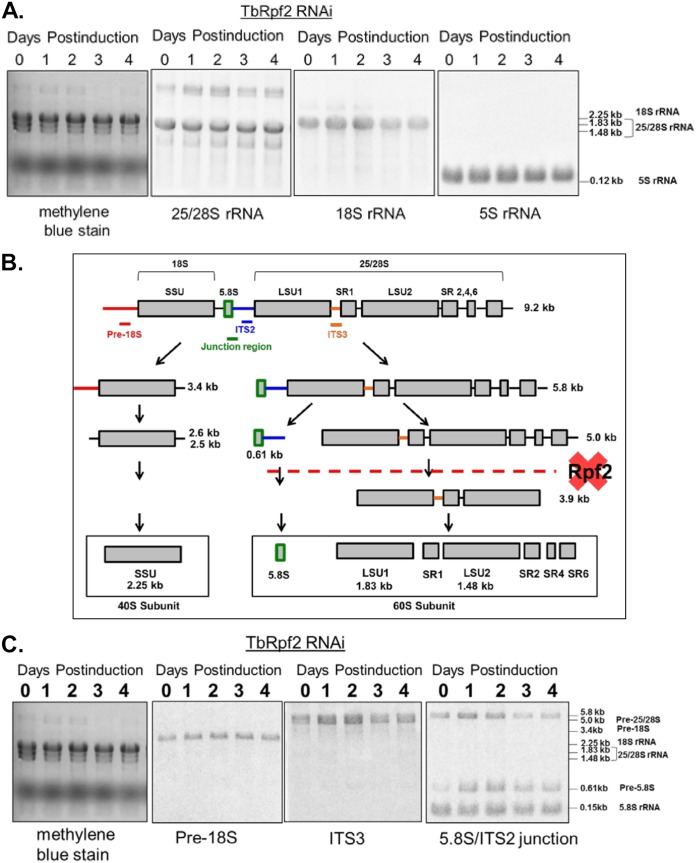
25/28S and 5.8S rRNA processing are disrupted by loss of TbRpf2. Total RNA was extracted from cells at days 0 to 4 of RNA interference induction as indicated. RNA was stained with methylene blue and then probed for various mature rRNAs (A) or rRNA processing intermediates (C) as labeled. Panel B is a map of rRNA processing altered to show the proposed site of processing interruption (38). Analyses were performed on three biological replicates, and representative blots are shown.

Defects in ribosome biogenesis are associated with improper processing of one or more rRNA intermediates. In T. brucei, rRNA processing exhibits unique peculiarities, specifically in the pathway leading to the formation of 25/28S rRNA, which is ultimately processed into six fragments (Fig. 4B). We began by analyzing the steady-state levels of mature rRNAs in both the TbRrs1 RNAi cell line (Fig. 4A) and a previously established TbRpf2 RNAi cell line (11) (Fig. 5A). We previously showed that loss of TbRpf2 disrupts 60S subunit formation, but the question of whether rRNA maturation was impacted was still unanswered. Evidence of partially processed intermediates was readily visible in methylene blue-stained membranes, so we next analyzed rRNA processing by probing total RNA for specific intermediate species throughout the time course of depletion. As shown in Fig. 4 and 5, intermediates in the generation of mature rRNA components of the 60S subunit accumulate in the course of induction. Specifically, the right arm of the pathway, including the 5.0-kb processing intermediate in the 25/28S branch as well as the 0.61-kb processing intermediate in the 5.8S branch, accumulates relative to uninduced cells (Fig. 4B and Fig. 5B). After 2 days of depletion of TbRrs1, the precursor-to-mature rRNA species ratios relative to day 0 are 6.1 for 25/28S rRNA (SD, 1.7), 9.3 for 5.8S rRNA (SD, 0.9), and 0.92 for 18S rRNA (SD, 0.4), In the case of TbRpf2 depletion, the precursor-to-mature rRNA species ratios relative to day 0 are 4.3 for 25/28S rRNA (SD, 0.9), 2.5 for 5.8S rRNA (SD, 0.1), and 1.1 for 18S rRNA (SD, 0.3). This further indicates that a loss of either TbRrs1 or TbRpf2 results in a defect in the generation of properly processed mature 60S particles.

### TbRrs1 is part of the 5S RNP and interacts with trypanosome-specific protein P34 and with TbL5.

**FIG 6:**
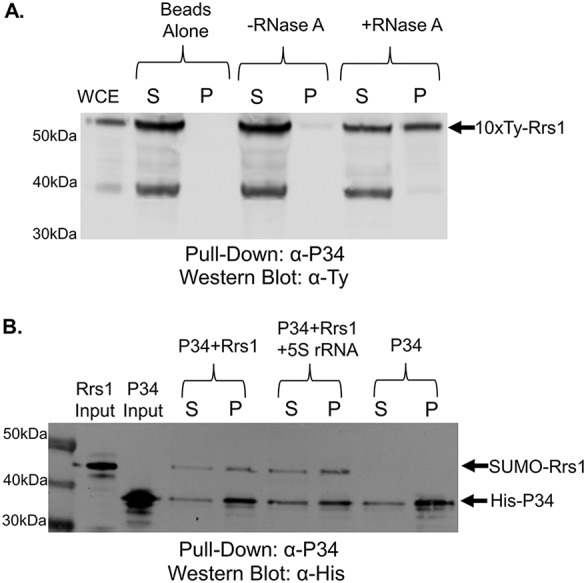
TbRrs1 interacts with P34/P37 *in vivo* and *in vitro*. (A) Whole-cell extract (WCE) was prepared from 10×-Ty-TbRrs1 cells, and coimmunoprecipitations were performed using anti-P34 antibody. The resulting immunoprecipitates were then analyzed via anti-Ty Western blotting. (B) Recombinant TbRrs1 was incubated with recombinant P34 in an anti-P34 coimmunoprecipitation assay with or without addition of *in vitro*-transcribed 5S rRNA. Western blot analyses were performed using anti-His antibody. Results shown are representative of three biological replicates. S, supernatant; P, pellet; WCE, 10 μg WCE.

We next performed immunoprecipitations to detect *in vivo* interactions between P34/P37, TbL5 or TbRpf2, and TbRrs1. We incubated whole-cell extract (Fig. 6A) with beads in the absence of antibody to check for nonspecific binding and observed no TbRrs1 in the control pellet fraction (Fig. 6A, Beads Alone). We then incubated whole-cell extract with beads conjugated to anti-P34/P37 antibody and observed an interaction between TbRrs1 and P34/P37 as seen in the pellet fraction (Fig. 6A, -RNase A). However, this interaction was significantly enhanced upon the digestion of cellular RNA through the addition of RNase A (Fig. 6A, +RNase A), as indicated by the increased TbRrs1 found in the pellet fraction (0.06 [SD, 0.03] to 0.61 [SD, 0.23] with addition of RNase A). This indicated that TbRrs1 and P34/P37 do interact *in vivo* and that cellular RNA plays a role in inhibiting the protein-protein interaction.

We next expressed SUMO-TbRrs1 and P34 (Fig. 6B, Rrs1 and P34 input), and when incubated together, the two proteins directly interact, as indicated by the presence of both proteins in the pellet fraction (Fig. 6B, P34+Rrs1). We then added *in vitro*-transcribed 5S rRNA to see if it altered the direct interaction but saw no significant impact upon the TbRrs1-P34 interaction (Fig. 6B, P34+Rrs1 + 5S rRNA, 0.77 [SD, 0.21] to 0.71 [SD, 0.23] after addition of 5S rRNA). This indicated that TbRrs1 and P34 directly interact and that 5S rRNA was not solely responsible for the strong inhibition of that interaction *in vivo*.

**FIG 7:**
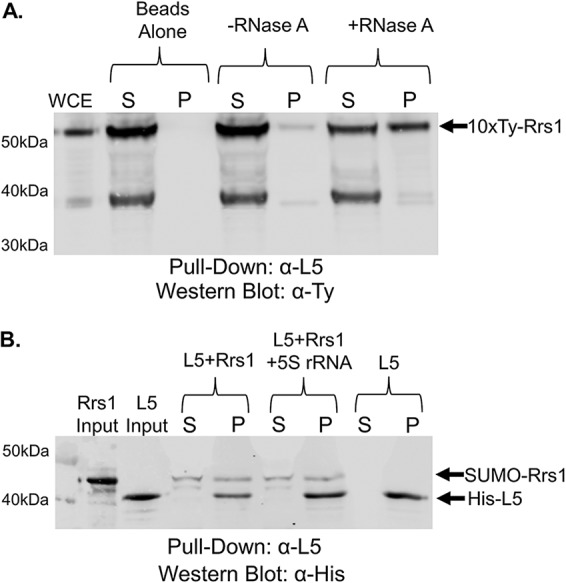
TbRrs1 interacts with TbL5 *in vivo* and *in vitro*. (A) Whole-cell extract (WCE) was prepared from 10×-Ty-TbRrs1 cells, and coimmunoprecipitations were performed using anti-TbL5 antibody. The resulting immunoprecipitates were then analyzed via anti-Ty Western blotting. (B) Recombinant TbRrs1 was incubated with recombinant TbL5 in an anti-L5 coimmunoprecipitation assay with or without addition of *in vitro*-transcribed 5S rRNA. Western blot analyses were performed using anti-His antibody. Blots shown are representative of three biological replicates. S, supernatant; P, pellet; WCE, 10 μg WCE.

We performed similar coimmunoprecipitation assays to examine the TbL5-TbRrs1 interaction. We saw a weak interaction between TbRrs1 and TbL5 (Fig. 7A, -RNase A) that was strongly enhanced (0.24 [SD, 0.28] to 0.63 [SD, 0.23] after addition of RNase A) by the digestion of cellular RNA (Fig. 7A, +RNase A). Using *in vitro* coimmunoprecipitation, we saw a direct interaction between TbL5 and TbRrs1 (Fig. 7B, L5+Rrs1) that was not impacted by the addition of 5S rRNA (Fig. 7B, L5+Rrs1 + 5S rRNA, 0.78 [SD, 0.18] to 0.74 [SD, 0.21] change after addition of 5S rRNA). Much as for P34, TbL5 and TbRrs1 interact *in vivo* and *in vitro*, and 5S rRNA is not solely responsible for the strong impact of cellular RNA on the L5-Rrs1 interaction.

### TbRrs1 directly interacts with TbRpf2 and forms a complex upon coexpression.

**FIG 8:**
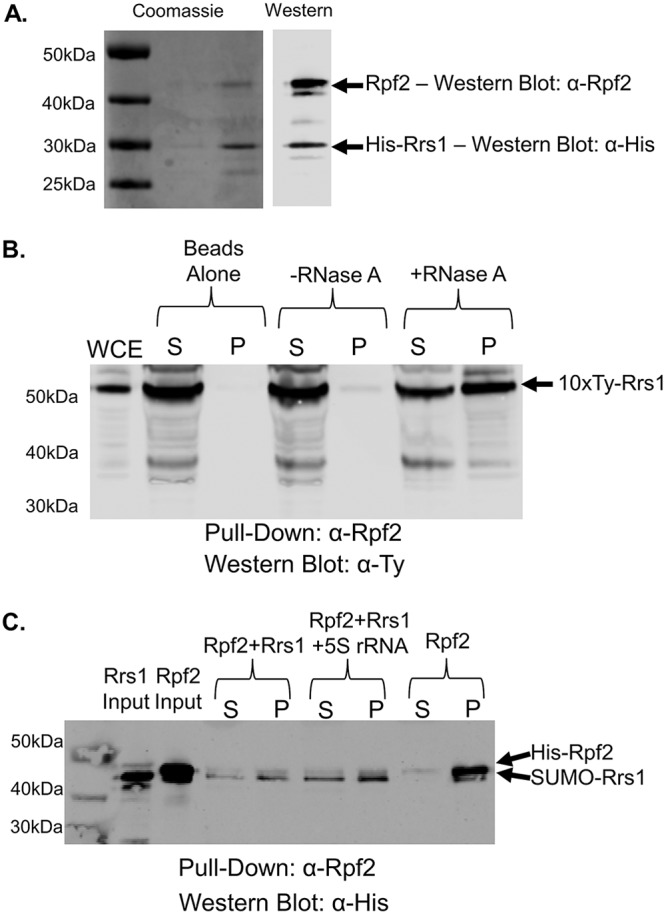
TbRrs1 interacts with TbRpf2 *in vivo* and *in vitro*. (A) Coomassie blue and anti-His/anti-Rpf2 Western blot analyses of the purification of coexpressed TbRpf2 and His-TbRrs1. (B) Whole-cell extract (WCE) was prepared from 10×-Ty-TbRrs1 cells, and coimmunoprecipitations were performed using anti-TbRpf2 antibody. The resulting immunoprecipitates were then analyzed via anti-Ty Western blotting. (C) Recombinant TbRrs1 was incubated with recombinant TbRpf2 in an anti-Rpf2 coimmunoprecipitation assays with or without addition of *in vitro*-transcribed 5S rRNA. Western blot analyses were performed using anti-His antibody. Results shown are representative of three biological replicates, and average values with standard deviations were calculated. S, supernatant; P, pellet; WCE, 10 μg WCE.

In yeast, Rrs1 and Rpf2 form a tight heterodimer that is required for S. cerevisiae Rpf2 stability and completion of the BRIX RNA-binding domain (13–15). In contrast, we have shown that TbRrs1 and TbRpf2 can be expressed independently from each other and that TbRpf2 retains its 5S rRNA binding capability (see above and reference 11). We generated a construct for simultaneous dual expression of TbRpf2-TbRrs1 and showed that purification based on the His-tagged TbRrs1 also copurified untagged TbRpf2 (Fig. 8A), indicating that in T. brucei, the two proteins form a strong complex.

We then used coimmunoprecipitations to confirm the TbRpf2-TbRrs1 interaction. TbRrs1-TbRpf2 weakly interacted *in vivo* before the addition of RNase A (Fig. 8B, -RNase A), after which the interaction increased significantly (Fig. 8B, +RNase A, 0.02 [SD, 0.01] to 0.58 [SD, 0.25] after addition of RNase A). Performing coimmunoprecipitation using recombinant TbRrs1 and TbRpf2, we observed a direct interaction (Fig. 8C, Rpf2+Rrs1) that was slightly increased by the addition of 5S rRNA (Fig. 8C, Rpf2+Rrs1 + 5S rRNA, 0.53 [SD, 0.18] to 0.65 [SD, 0.19] after addition of 5S rRNA). Therefore, despite the stability of both TbRpf2 and TbRrs1 when expressed independently *in vitro*, they are also able to tightly associate under multiple conditions and the association is slightly enhanced by the presence of 5S rRNA.

### TbRrs1 directly binds to 5S rRNA but does not impact TbRpf2 binding to 5S rRNA in T. brucei.

**FIG 9:**
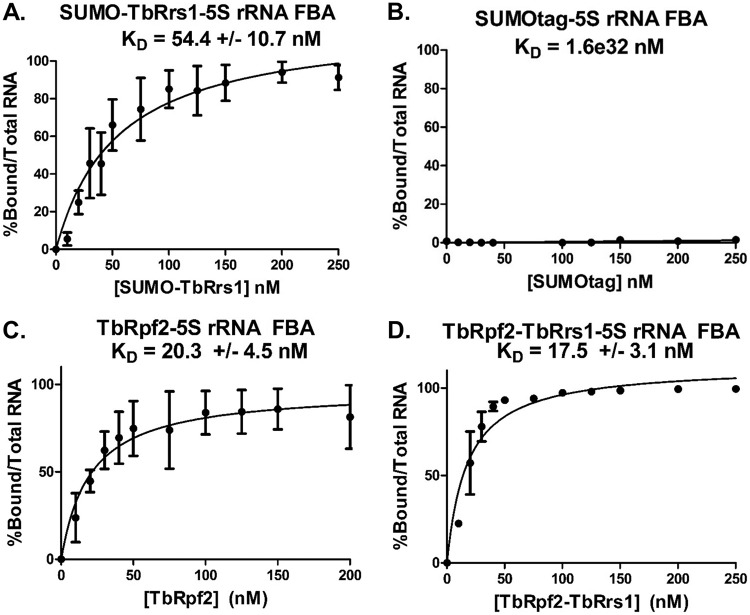
TbRrs1 directly binds to 5S rRNA but does not impact TbRpf2 binding to 5S rRNA in T. brucei. Increasing concentrations of recombinant SUMO-TbRrs1 (A), SUMO tag (B), (His)_6_-TbRpf2 (C), or TbRpf2-(His)_6_TbRrs1 (D) were used in filter binding assays (FBAs) with radiolabeled 5S rRNA to determine a binding curve of the protein-RNA interaction. Analyses were performed in triplicate, and average values with standard deviations were calculated.

We next examined if TbRrs1 was also able to bind to 5S rRNA independently. We determined that recombinant SUMO-tagged TbRrs1 directly binds to 5S rRNA with a *K_D_* (equilibrium dissociation constant) of 54.4 (SD, 10.7 nM) using filter binding assays (Fig. 9A). Significantly, the SUMO tag alone does not directly interact with 5S rRNA (Fig. 9B), indicating that the TbRrs1-5S rRNA interaction was due to TbRrs1. These results clearly show that TbRrs1 has 5S rRNA binding capabilities in the absence of TbRpf2, a property that has not been shown for any other studied Rrs1 (14, 15).

We next showed that the coexpressed TbRpf2-TbRrs1 directly interacts with 5S rRNA with a *K_D_* of 17.5 (SD, 3.1) nM (Fig. 9D). This is very similar to the strength of the interaction of TbRpf2 and 5S rRNA in the absence of TbRrs1 at 17.0 (SD, 4.4) nM under the same conditions (Fig. 9C) (11), suggesting that the ability of TbRrs1 to bind 5S rRNA on its own does not strongly impact the interaction of TbRpf2 with 5S rRNA.

### TbRrs1 is structurally different from yeast and other eukaryotes.

**FIG 10:**
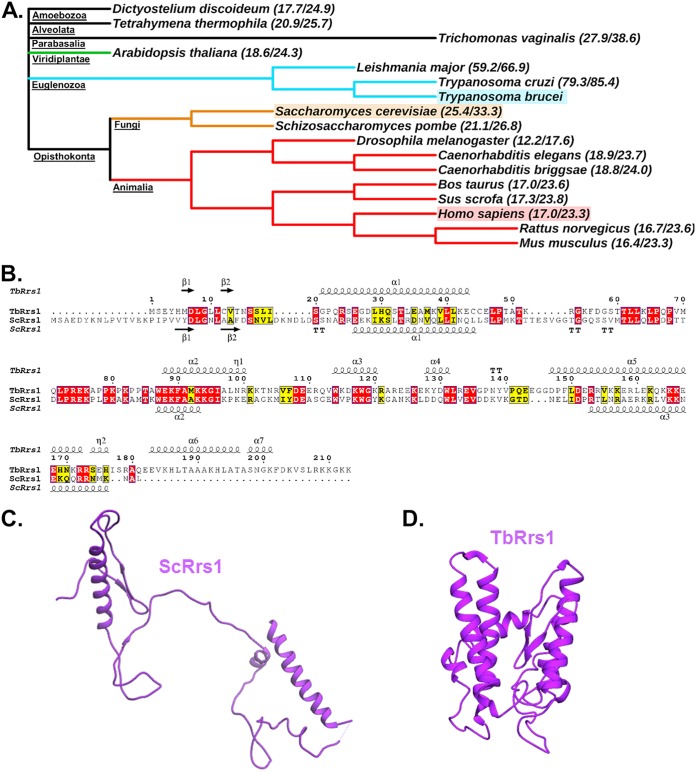
The predicted model of TbRrs1 is structurally different from yeast and other eukaryotic organisms. (A) Cladogram depicting the levels of evolutionary separation between T. brucei, Homo sapiens, and S. cerevisiae, the model yeast. Percent identity and similarity of Rrs1 with T. brucei are presented beside each organism. (B) Primary and secondary structural alignment of T. brucei versus S. cerevisiae Rrs1. (C and D) Published cryo-electron microscopy tertiary structure of S. cerevisiae (C) and a predicted structure for TbRrs1 (D).

We next compared the sequences of TbRrs1 to homologues in other eukaryotes (Fig. 10A). The S. cerevisiae homologue is only 25.4% identical and 33.3% similar to TbRrs1. Even Rrs1 from Leishmania major (a closely related kinetoplastid parasite) showed only 59.2% identity and 66.9% similarity to TbRrs1, suggesting very different characteristics even among closely related organisms. We next performed *in silico* predicted modeling of TbRrs1 to compare secondary (Fig. 10B) and tertiary (Fig. 10D) structures with the S. cerevisiae homologue. Interestingly, some of the secondary structural features aligned well between the two proteins at their N-terminal regions (Fig. 10B), particularly two beta-strands and an alpha-helix that complete the BRIX domain in ScRpf2 (13–15), and these features seem to be conserved across organisms (data not shown). However, TbRrs1 has a greater structural divergence toward its C terminus, which features many more predicted alpha helices and a longer C terminus as a whole. This resulted in a more compact predicted structure (Fig. 10C and D), which may explain the differing characteristics of TbRrs1 and ScRrs1 identified in this study.

## DISCUSSION

Eukaryotic ribosome biogenesis is essential for all organisms, and much of our understanding of this process comes from studies performed in S. cerevisiae. However, while this and other model organisms have been helpful in elucidating the complexities of ribosome biogenesis, they also narrow our understanding, missing the potential diversity that could exist in other eukaryotic systems. Recent publication of high-resolution structures of ribosomal subunits from Leishmania donovani (20), Trypanosoma cruzi (21), and Trypanosoma brucei (22) have highlighted how different this highly conserved process may be outside S. cerevisiae. The presence of large expansion segments in both protein and rRNA components and of additional points of cleavage in the 25/28S rRNA suggests the need to further explore the unique characteristics of ribosome biogenesis in diverse organisms.

We have focused on the assembly and incorporation of the 5S RNP to identify trypanosome-specific features of this critical part of ribosome biogenesis, including the presence of the trypanosome-specific proteins P34/P37 as members of that complex. These proteins directly interact with two other 5S RNP proteins, TbL5 (23) and TbRpf2 (11), as well as the 5S rRNA (9, 24). Furthermore, we have shown that P34/P37 are essential and that their loss impacts 5S rRNA abundance and ribosome biogenesis. We have also identified unique characteristics in TbL5 (23) and TbRpf2, which are essential in T. brucei, and have roles in ribosome biogenesis. Of particular interest, we found that TbRpf2 could be expressed *in vitro* as a functional protein in the absence of TbRrs1, a characteristic that was not observed in studies of yeast Rpf2. We were therefore interested in examining how the T. brucei homologue of Rrs1, an additional protein member of the 5S RNP in yeast, might differ in this organism.

We began by showing that loss of TbRrs1 (Fig. 1B) resulted in a strong growth defect (Fig. 1A) and in aberrant morphology (Fig. 1C). Thus, TbRrs1 is essential for T. brucei growth and survival. We next observed that loss of TbRrs1 resulted in a decrease in levels of its binding partner TbRpf2 (Fig. 2) but not in other members of the 5S RNP, P34/P37 and TbL5 (Fig. 2). This suggests it might have an important shared role with TbRpf2 but that there is no significant interdependence between levels of TbRrs1 and the other proteins in the 5S RNP.

We next examined the impact that loss of TbRrs1 has on ribosomal subunit formation and translation as an indicator of ribosome biogenesis dysfunction. Polysome profiles following depletion of TbRrs1 showed a reduction of the 60S and polysome peaks, the formation of halfmers (19), and an increase in monosomes and 40S peaks (Fig. 3). At the level of mature rRNA, there was a general decrease in 25/28S rRNA but no change in 18S rRNA or 5S rRNA (Fig. 4A). Furthermore, we saw an increase in the 5.8- and 5.0-kb intermediates of the 25/28S pathway and in the 0.61-kb intermediate of the 5.8S pathway (Fig. 4C). The combination of the polysome profiles and the rRNA processing Northern blots demonstrates that TbRrs1 plays a critical role in 60S biogenesis in T. brucei.

For comparison, we also used a TbRpf2 RNAi line (11) which we had previously shown disrupts 60S subunit biogenesis upon induction of RNAi knockdown. Loss of TbRpf2 resulted in an increase in 5.0-kb and 5.8-kb intermediates (part of the 25/28S rRNA pathway) and the 0.61-kb intermediate (part of the 5.8S rRNA pathway) (Fig. 5C). These findings are consistent with observations from polysome profiles that loss of TbRpf2 disrupts 60S biogenesis (11), suggesting that disruption occurs at both the subunit assembly and rRNA processing levels. There is no significant change in the levels of mature 18S or 5S rRNA, but also not in the levels of mature 25/28S rRNA, an observation previously found in the literature (25). This could partially be accounted for by kinetic effects, where processing is delayed, leading to longer half-lives of intermediates that become readily detectable, while maintaining bulk synthesis through the pathway. These experiments showed that TbRrs1 is an essential part of 60S biogenesis in T. brucei and that while losses of TbRrs1 and TbRpf2 have similar impacts on 25/28S rRNA processing and 5.8S rRNA processing, they may differ in the extent to which the kinetics of processing is altered, leading to different effects on steady-state levels of mature rRNAs. Furthermore, while there are unique aspects to rRNA processing in T. brucei, this disruption of rRNA processing is similar to that observed with loss of Rrs1 (16) and Rpf2 (17) in S. cerevisiae, further strengthening a role for TbRrs1 and TbRpf2 in this essential process.

We next addressed whether TbRrs1 directly interacts with other members of the 5S RNP and is a member of the complex. We saw that P34/P37 and TbRrs1 interact weakly *in vivo* but that degradation of cellular RNA strengthens this interaction (Fig. 6A). This would suggest that RNA is involved in competing with or disrupting this interaction. However, using recombinant protein we saw that the addition of 5S rRNA did not change the direct interaction between TbRrs1 and P34/P37 (Fig. 6B). Next, we looked at the TbL5-TbRrs1 interaction and saw that, much like with P34, *in vivo* TbL5 and TbRrs1 had a weak interaction that was significantly strengthened by loss of cellular RNA (Fig. 7A). However, 5S rRNA did not impact the direct *in vitro* interaction of TbL5 and TbRrs1 (Fig. 7B). This suggests that the dramatic increase in binding between TbRrs1-P34 and TbRrs1-TbL5 seen with degradation of cellular RNA is likely due to RNA other than 5S rRNA.

In yeast (13–15) and Arabidopsis thaliana (26), Rpf2 and Rrs1 form a tight heterodimer. Furthermore, in S. cerevisiae, it has been shown that Rpf2 and Rrs1 generally bind only to 5S rRNA as the heterodimer, since Rrs1 is required for *in vitro* Rpf2 stability (14). We have previously shown that TbRpf2 can be purified and bind to 5S rRNA independently (11). We have shown here that TbRrs1 can be purified independently and that it forms stable interactions with other 5S RNP proteins. We next found that TbRpf2 copurified with TbRrs1 (Fig. 8A), suggesting that the two proteins form a tightly bound, stable heterodimer. TbRrs1 and TbRpf2 also weakly interacted in *in vivo* coimmunoprecipitations, and this was strongly enhanced by the degradation of cellular RNA (Fig. 8B). Furthermore, the direct interaction between TbRrs1 and TbRpf2 observed *in vitro* was slightly increased by the addition of 5S rRNA (Fig. 8C). Taken together, these data suggest that the two T. brucei proteins form a complex despite the ability to purify each independently, indicating that the heterodimer is not required for protein stability.

These results indicated that TbRrs1 interacts with the protein members of the 5S RNP in a largely RNA-dependent fashion. Previous work in S. cerevisiae had shown that while it could not be purified independently, Rpf2, but not Rrs1, was able to directly interact with 5S rRNA (14, 15). We demonstrated that TbRrs1 expressed independently from TbRpf2 directly binds to 5S rRNA (Fig. 9A), in contrast to yeast, where no direct binding was seen for ScRrs1 and 5S rRNA (14, 15). Dually expressed TbRpf2-TbRrs1 also directly interacted with 5S rRNA (Fig. 9D) with a *K_D_* very similar to that of TbRpf2 alone (Fig. 9C) (11). This suggests that despite the novel interaction between TbRrs1 and 5S rRNA, it does not result in noticeably increased binding capacity of the resulting heterodimer. This suggests that while the incomplete BRIX domains of the two separate proteins are able to effectively bind 5S rRNA, the capacity of the combined proteins to bind 5S rRNA is not increased. Rather, the role of TbRrs1 in completing this complex might have an important function beyond the act of RNA binding. This would be consistent with structural data in S. cerevisiae which suggested that Rrs1 might act to anchor Rpf2 and other 5S RNP proteins to the ribosome through interactions occurring at the Rrs1 C terminus (27). In the context of ribosome biogenesis, TbRrs1 may help anchor TbRpf2 and the remaining 5S RNP components to the ribosome during the 5S rRNA conformational shift. TbRpf2-TbRrs1 could be disrupted by further steps in ribosome maturation, liberating TbRrs1 both from the nascent ribosome and from TbRpf2. This could free up TbRpf2 and TbRrs1 for nonribosome biogenesis roles in the cell, consistent with some observations in yeast that TbRrs1 plays a role in chromosome congregation (28) and, in humans, a role in Huntington disease (28, 29). This same explanation could also apply to the interaction of TbRrs1 with trypanosome-specific P34/P37, which we have previously shown to have an important additional role in 60S subunit export (30).

Given these observed functional differences in TbRrs1, we examined its relative similarity to other eukaryotic homologues. There was a great deal of variation among the eukaryotic homologues of Rrs1, even within the *Trypanosomatidae* (Fig. 10A and B). Despite some similarity in secondary structure at their N termini, which contains the elements necessary for completing the BRIX domain and thus suggesting some structural and possibly functional similarity, the T. brucei and S. cerevisiae Rrs1 proteins differ significantly toward their C termini. In T. brucei, the Rrs1 C terminus is longer, containing more numerous and extensive predicted alpha helices. These differences are made more evident in the dissimilarities seen in a comparison of their tertiary structures (Fig. 10C and D). The more compact and complex structure of TbRrs1 could be important to its unique characteristics, including the novel interactions in the 5S RNP, especially its binding to 5S rRNA, and might help explain the differences that set it apart from its homologue in yeast.

While model organisms are immensely informative when it comes to understanding the details of complex processes, limiting studies to a few model organism prevents an understanding of how the process might vary across diverse organisms. Eukaryotic ribosome biogenesis is a very complex process that has largely been studied in the yeast S. cerevisiae. Ongoing work in T. brucei has highlighted how certain steps of ribosome biogenesis, such as export (30), rRNA processing (20, 31), and the formation and incorporation of the 5S RNP (10, 11, 23), can vary greatly between different organisms. In this study, we have begun the characterization of a T. brucei homologue of the 5S RNP protein Rrs1 and examined in detail how it might differ from what is known about the properties of the yeast Rrs1. We found that TbRrs1 was essential for parasite survival and that, as a crucial member of the 5S RNP, it not only interacted with other 5S RNP members but also had an important role in ribosome biogenesis and, along with TbRpf2, rRNA processing. This highlights some of the features that are similar between the T. brucei and S. cerevisiae Rrs1 proteins, but we found additional trypanosome-specific characteristics of TbRrs1. Two clear examples of the dissimilarity are that TbRrs1 interacted with the trypanosome-specific proteins P34/P37 and that it was able to directly bind to 5S rRNA. Furthermore, although TbRrs1 and TbRpf2 form a heterodimer, both TbRrs1 and TbRpf2 can be expressed and bind 5S rRNA independently, suggesting both separate and combined functions. This work both expands our understanding of the complex process of ribosome biogenesis and highlights unique features present in pathogens that may allow targeting of ribosome biogenesis for future drug development.

## MATERIALS AND METHODS

### Generation of RNAi and Ty-tagged cell lines.

Primers were used (Table 1) to PCR amplify the full TbRrs1 gene product (Tb927.06.2050), and the product was simultaneously digested and ligated into p2T7-177 expression plasmid (32). The resulting plasmid was linearized and transfected (Amaxa Nucleofector II) into the procyclic 29-13 strain (33, 34), and cells were selected with phleomycin (2.5 μg/ml). Growth curves were calculated in the presence or absence of tetracycline (2.5 μg/ml) and are based on three biological replicates, with average values and standard deviation shown.

**TABLE 1:** Primer sequences

Primer/purpose	Sequence
TbRrs1 Bam For/RNAi construct	5′-CAC CAC AGC CAG GAT CCG ATG AGT GAG TAT CAC A
TbRrs1 Hind Rev/RNAi construct	5′-TAT GCG GCC GCA AGC TTT TAC TTT TTA CCC TTC
10×-Ty-TbRrs1 PCR For	5′-TTCATCTTATACTTCTATTCACTTTTATCCCTCGTACCCCCTGTTTGTAGGGCACTCACGTAGGTTAACAGGACCGAAGAGT AAATGCAGACCTGCTGC
10×-Ty-TbRrs1 PCR Rev	5′-CCCTCGCTCCGCTGCGGTCCACTAATCAGAGAGCTGTTCGTCACGCAAAGAAGCCCCAGATCCATGTGATACTCACTCA TATCCAAGGGATCTTGATT
TbRrs1 For/pET-SUMO construct	5′-ATG AGT GAG TAT CAC ATG GAT CTG
TbRrs1 Rev/pET-SUMO construct	5′-TTA CTT TTT ACC CTT CTT CCT GAG
TbRpf2 Nde For/pET-DUET construct	5′-CAG CAG GAG ATA TAC ATA TGT CCT CTA TCG GTG
TbRpf2 Kpn Rev/pET-DUET construct	5′-GCC AAT CGA GCG GTA CCT CAA ATA TCC CTA TCG
TbRrs1 Bam For/pET-DUET construct	5′-CAC CAC AGC CAG GAT CCG ATG AGT GAG TAT CAC A
TbRrs1 Hind Rev/pET-DUET construct	5′-TAT GCG GCC GCA AGC TTT TAC TTT TTA CCC TTC

10×-Ty-tagged cell lines were prepared in either wild-type 427 cells or the TbRrs1 RNAi cell line as previously described (Table 1) (plasmids generously provided by Sam Dean) (35). Cells were selected using blasticidin, and clonal lines were prepared via extreme limiting dilution. Tagged cell lines were used to determine the degree of knockdown of Rrs1 and *in vivo* coimmunoprecipitation since we were unable to generate a specific antibody for TbRrs1. Growth levels of tagged and untagged cell lines were equivalent, as were expression levels of 5S RNP and S5 proteins.

### Western blots.

Whole-cell lysate was prepared as previously described (11). Fifteen micrograms of extract was transferred to an 0.4-μm nitrocellulose membrane (Bio-Rad) and probed using antibodies for TbL5 (10) and P34/P37 (36) at dilutions of 1:1,000, anti-Ty (ThermoFisher) at a dilution of 1:2,000, HSP70 (37) at a dilution of 1:20,000, and TbRpf2 (11) and anti-S5 (Abnova) at dilutions of 1:500 in Odyssey blocking buffer (Li-Cor Technologies). For *in vitro* coimmunoprecipitations, anti-His (ThermoFisher) was used at a dilution of 1:1,000 in Odyssey blocking buffer (Li-Cor Technologies). Li-Cor secondary antibodies were used to allow quantification of the signals in the Image Studio software (Li-Cor Technologies), as they are directly proportional to the amount of target protein. All analyses shown are representative blots from three biological replicates, and data were calculated relative to HSP70, compared to uninduced cells, and presented as averages with standard deviations.

### DIC microscopy.

Both wild-type and TbRrs1 cells were prepared for differential interference contrast (DIC) microscopy as previously described by fixation followed by mounting using Prolong Gold Antifade reagent with 4′,6-diamidino-2-phenylindole (DAPI) (Life Technologies) and imaged using a Zeiss Axioimager M2 microscope and the Volocity 6.1 Acquisition software. Experiments were performed in triplicate with representative results being shown.

### Polysome profiles.

Polysomes, monosomes, and ribosomal subunits were isolated from 5 × 10^8^ cells by ultracentrifugation on 10 to 40% sucrose gradients as previously published (7). Profiles were examined at the days indicated, with a representative sample from three biological replicates shown.

### Total RNA extraction and mRNA Northern blots.

Total RNA was extracted using TRIzol according to the manufacturer’s instructions, and Northern blot analysis was performed on 5 μg of that RNA as previously described (38). Images were captured using a Typhoon phosphorimager (GE Technologies) and quantified using the ImageJ software (39). All analyses are presented as representative of three biological replicates.

### Cloning and expression of recombinant proteins.

N-terminal SUMO-TbRrs1 (Tb927.06.2050) was prepared by amplification of the full gene products from T. brucei genomic DNA with the primers indicated (Table 1), ligated into the Champion pET-SUMO vector (Life Technologies), and expressed in Escherichia coli BL21(DE3)Star One Shot cells (Life Technologies). Purification was performed as previously described (10), and proteins were detected via Western blotting using anti-His.

A dual-expression TbRpf2-(His)_6_TbRrs1 construct was generated by amplification of the full gene products from T. brucei genomic DNA (Table 1) and inserted into pET-DUET1 vector. The final construct was transformed into E. coli BL21(DE3)Star One Shot cells for expression and purified as described above, and proteins were detected via Western blotting using anti-His or anti-Rpf2 antibody.

### Coimmunoprecipitations.

Coimmunoprecipitation was performed with either whole-cell lysate from Ty-tagged Rrs1 cells or recombinant proteins as previously published (40). P34 was solely used *in vitro* as representative of P34 and P37, since we have previously shown that they are functionally identical (9, 10). Bound protein was eluted, and the supernatant containing unbound protein was ethanol precipitated followed by resuspension in 1× SDS sample buffer. The entire eluted pellet fraction and the ethanol-precipitated supernatant were electrophoresed and blotted as described above. All analyses are presented as representative blots from three biological replicates, and data were quantified using Image Studio (Li-Cor Technologies) as amount in the pellet relative to the total protein in the pellet and supernatant fractions and presented as calculated means with standard deviations.

### *In vitro* transcription of 5S rRNA.

The 5S rRNA was *in vitro* transcribed as described previously (9) and treated with DNase I (Life Technologies) to remove template DNA, and proteins and unincorporated nucleotides were removed using NucAway spin columns (Ambion).

### Filter binding assay.

Radiolabeled 5S rRNA was prepared as described above, and filter binding assays were performed as previously published (9). Membranes were analyzed with a Typhoon phosphorimager (GE Healthcare), and binding affinity values were calculated using Quantity One (Bio-Rad) and Prism (GraphPad) software as previously described (41). All analyses were taken from three biological replicates with average values and standard deviations calculated in Prism (GraphPad) and presented above.

### TbRrs1 structural modeling and analyses.

Primary and secondary structural information was obtained from UniProt where available, with some secondary and tertiary information being retrieved from RCSB PDB files and some predicted using the iTasser software (42). Sequences were aligned using Clustal Omega (43), and identities and similarities were calculated using online server software from SIAS (http://imed.med.ucm.es/Tools/sias.html) and SMS (http://www.bioinformatics.org/sms2/ident_sim.html). Taxonomic trees were designed using NCBI Common Tree, NCBI Tree Viewer, and Interactive Tree of Life (44). All sequence alignments and corresponding secondary structural elements were created using ESPript (45), and all tertiary structural components were modeled using Chimera (46).
